# How Does Fiction Reading Influence Empathy? An Experimental Investigation on the Role of Emotional Transportation

**DOI:** 10.1371/journal.pone.0055341

**Published:** 2013-01-30

**Authors:** P. Matthijs Bal, Martijn Veltkamp

**Affiliations:** 1 Department of Management & Organization, VU University Amsterdam, Amsterdam, The Netherlands; 2 FrieslandCampina, Deventer, The Netherlands; Boston College, United States of America

## Abstract

The current study investigated whether fiction experiences change empathy of the reader. Based on transportation theory, it was predicted that when people read fiction, and they are emotionally transported into the story, they become more empathic. Two experiments showed that empathy was influenced over a period of one week for people who read a fictional story, but only when they were emotionally transported into the story. No transportation led to lower empathy in both studies, while study 1 showed that high transportation led to higher empathy among fiction readers. These effects were not found for people in the control condition where people read non-fiction. The study showed that fiction influences empathy of the reader, but only under the condition of low or high emotional transportation into the story.

## Introduction

Reading books and watching movies, plays, and operas are activities that people carry out on a day-to-day basis in their lives. Activities like these are referred to as the experience of *fictional narratives* [1], [2], and they may provide people with distraction from daily demands and possibly initiate intellectual inspiration [3]. Fictional narrative experience may have an important and profound impact on how people feel and behave in their daily lives [4]. For instance, it has been suggested that fictional narratives provide personal insights, and therefore are important for people in order to learn about themselves [2], [3]. One direction that research on the effects of fiction experience has taken is whether fiction experience influences empathy of the reader [5]–[7]. It has been suggested that people who read a lot of fiction become more empathic, because fiction is a simulation of social experiences, in which people practice and enhance their interpersonal skills [3]. However, although studies have shown that fiction is correlated with empathy, there are several shortcomings to previous research.

First, researchers have questioned the causal relationships between experience of fiction and empathy. Does the experience of fiction really lead to higher empathy, or is it that highly empathic people tend to read more fiction, and therefore fiction is positively associated to empathy, as Argo et al. [8] have suggested? In other words, empathic people might simply enjoy fiction reading, and therefore the two are positively related to each other, excluding the possibility to draw conclusions about causal relations between fiction reading and empathy. A strict test of this question requires an experimental design in which effects of fiction experience over time can be assessed. Second, there have been no studies where effects of fiction reading on empathy are investigated using real existing stories. Until now, research designs have been based on either proxies of experience of fiction (e.g., knowledge of fiction authors) [6]–[7] or on very short texts that participants in experiments have to read [9], [10], limiting the ecological validity of studies on the effects of fiction on empathy. Therefore, it is imperative that the effects of fiction reading on empathy are investigated under realistic conditions in an experimental design, in order to rule out reversed causality in the relationships [5]. There have been very few studies that have investigated effects of fiction over time. The current study addresses these limitations of earlier research by presenting two experimental investigations of the relationships between fiction experience and empathy, while comparing these relations to a control condition where people read non-fiction.

Finally, the study investigates the role of emotional transportation [11] in the aforementioned relationships. We propose that fiction experiences will change an individual’s empathic skills only when the reader is emotionally transported in a story, as suggested by Oatley [3]. By looking at the moderating role of transportation [11]–[14], we investigate the assumption that people’s empathic skills will only be enhanced when the reader becomes emotionally transported by a fictional narrative. Although researchers have mentioned the role of transportation, there are very few studies that have empirically tested the influence, and until date, no study has looked at the role of transportation in predicting empathy.

The current article presents two experiments on the effects of fiction reading on empathy, and thereby makes several contributions to the existing literature. Through two empirical investigations of actual experience of literature reading (compared to a control condition), through studying the effects of fiction experience over time whilst controlling for previous levels of empathy and experienced negative and positive emotions during reading, and finally through investigation of the conditions under which fiction leads to changes in empathy (through looking at the moderating role of transportation), this study contributes to the field of investigation of effects of fictional narrative experience, and provides an answer to the question whether actual fiction experience influences individuals [7].

### Fiction, Non-fiction and Narrative Structures

It has been argued that fiction may elicit stronger emotional and behavioral effects than nonfiction reading (e.g., newspapers and nonfiction books) [15]. Hence, a difference can be made between fictional narratives and non-fictional writing. Bruner [16] argued that narrative cannot be separated from fiction because every narrative told by an individual includes an interpretation of an event, and the narrator’s goals in telling the story. Hence, the difference between fiction and non-fiction is difficult to establish [16], and the narrative structure of the text determines the extent to which the text is able to influence a reader. Bruner, however, distinguished logico-scientific mode of thinking and the narrative mode. While the first is aimed at seeking universal truth conditions through argumentation and logic, which can be represented by for instance scientific publications and newspapers (henceforth nonfiction), the narrative mode aims at particular truth conditions, and establishes verisimilitude, or truthlikeness. The central focus of the narrative mode is believability, as assessed by the reader. This narrative mode of thinking is best represented by fictional literature [17]. Fiction focuses on believability; a fictional text is not assessed on its consistency as is the case in non-fiction, but rather on whether it establishes verisimilitude, or truthlikeness [16], [18]. A reader will be affected by a fictional narrative only when it creates a narrative world that is real within its context, and more importantly, when it is realistic for the reader, thereby creating an opportunity to be drawn into the story, which is discussed in more detail later on. However, nonfictional logico-scientific thinking will not be able to elicit those feelings [16], [19]. Fictional narratives present characters, events and the setting of a story in such a way that the reader can become transported and hence change through the narrative [17], [20].

### Effects of Fiction Experience on Empathy

Even though little research has been conducted on the effects of fiction reading on empathy, there are several researchers who have explained why fiction reading influences empathy. Mar and colleagues [6], [7], [21] argued that fiction reading may have profound effects on empathic skills of the reader. When an individual reads a story, emotions are triggered by that story, such that an affective impression is elicited by the narrative. According to Oatley [2], fiction presents a simulation of real-world problems, and therefore has real consequences for the reader. Often when someone reads a fictional story, identification with the characters in the story and emotional involvement in the story causes the reader to sympathize with the characters, and perhaps even experience the events in the story as if the reader experiences the events him−/herself. Consequently, the reader practices being empathic while reading a fictional story. We define empathy in line with Davis [22], [23] as: the cognitive and intellectual ability to recognize the emotions of other persons and to emotionally respond to other persons [24]. It includes sympathy and concern for unfortunate others [23]. Study of empathy is important because high empathic persons are more prosocial which is associated for example in the workplace to higher performance, productivity, and creativity [25], [26]. There are several reasons why fiction reading may be related to empathic skills.

First, the simulation of real-world experiences in fiction might be associated with processes that people use in daily life to comprehend what happens in the world [7]. Consequently, through this sensemaking process, people gain a better understanding of the world and how they should interact with other people. People learn from fiction about the human psychology, and gain knowledge about how to react to other people in social situations, as argued by Mar et al. [7]. When an individual reads a story, he/she predicts the actions and reactions of the characters, by inferring what they are thinking, feeling, and intending. In order to do this, the reader sympathizes with the characters in the story, through taking the perspective of the characters and to experience the events as if it is the reader’s own experience. Moreover, some stories are able to make sense out of the senseless, and offer possibilities to understand other people across time and space, an opportunity which is not readily available in daily life [27]. The sympathy a reader feels for the characters is then integrated in the self-concept of the reader, through which the reader accumulates his/her ability to take the perspective of others, and to feel empathy [28]. Moreover, enhancement of empathic skills through fiction reading can contribute to people’s goals of who they want to be in their lives, such as to become a person that cares for other people’s welfare [29]. Hence, sympathetic reactions to fictional characters are integrated into broader response patterns in daily life, and empathic skills of the reader are enhanced [30].

Second, Mar et al. [6] argued that fiction experiences enhance imaginative thinking. In line with the Immersed Experiencer Framework [20], fictional words and stories activate neural processes that reflect real-world events which are similar to the story. Zwaan [20], [31] introduced the Immersed Experiencer Framework to explain language comprehension by three mechanisms. When an individual reads a text, neural webs are activated while reading, through which an event in a story is simulated mentally by the reader. Finally, the reader integrates that what is read with existing mental models. Hence, this model explains at the language comprehension level that readers actively process texts and integrate these texts in their own human experiences [20]. Indeed, there is evidence suggesting that seeing or reading about another person experiencing specific emotions and events activates the same neural structures as if one was experiencing them oneself, consequently influencing empathy [32]. Thus, by reading a story, people imagine a narrative world that is similar to our own world. In this narrative world, people imagine how it is to see through the eyes of other people, by imagining and actually experiencing the thoughts and feelings of characters in a story. Hence, imaginative processes, evoked by fictional narrative experience, make people more empathic. Consequently, we argue that the reader becomes more empathic while reading fiction. The question however, is why fiction has such a potential impact on people.

### Why does Fiction have an Impact on our Lives?

Fiction is primarily aimed at eliciting emotions [2], [3]. To become engaged in a fictional story, a reader suppresses the notion of fictionality of the story and the characters to experience the emotions of the characters [15]. According to Goldstein [15], a person reading fiction tends to react more strongly towards a story than when he/she would read a non-fictional story, because fiction provides a safe arena in which a reader can experience emotions without the need for self-protection. Because fiction does not follow the reader into real life, the reader can allow oneself to freely experience strong emotions, without immediate transfer of these emotions to real life. Moreover, we can allow ourselves to sympathize strongly with a character of a fictional story, because we do not have obligations towards the characters of a fictional story, while sad reports in a newspaper may cause feelings of obligation towards the victims to help them.

Another reason why fiction may have stronger effects on empathy than nonfiction is that fiction is processed differently than communications that aim to persuade a reader, such as commercial messages, scientific articles, opinion articles in newspapers, et cetera [33], [34]. The effects of persuasive communication are likely to diminish over time, unless people are highly motivated and hence process the information in a systematic and elaborative way, in line with the Elaboration Likelihood Model [35]. For instance, a message about the negative effects of smoking may only temporarily change the beliefs of a reader. However, research has shown that individuals may be strongly influenced when they read fictional stories [34], [36], [37]. While readers are likely to read critically within the context of persuasive communication, a fictional narrative is more likely to be read with a willing construction of disbelief: the readers accepts assertions from a fictional narrative unless the reader is highly motivated to reject the assertion and is able to reject the assertion based on available knowledge [36], [38]. Hence, the possible effects of stories on empathy are expected to be greater for fiction readers than for non-fiction readers.

Finally, another reason why nonfiction may have less strong effects on empathy than fiction has been presented by the theory of psychic numbing [39]. Slovic argues that the way a message (e.g., about victims) is presented to people influences their capacity to experience the affective information in that message and to feel sympathy. Specifically, it is easier to experience affect if a message presents information about a single, identifiable individual, than when information is presented about entire groups or using statistics (i.e., you can place yourself in the shoes of one other, but not of thousands). As a result, it has been shown in research on donating behavior that people will donate more money after reading information about an identifiable individual that suffered (e.g., one individual faces hunger) than after reading a message showing group statistics (e.g., 3 million people face hunger) [40]. In other words, a process of psychological numbing towards stories about large groups of people or objectified or statistically presented facts (which are often presented in non-fiction such as newspapers) is likely to occur, while fictional narratives, which are characteristically about individuals and their personal stories, may influence people to a much stronger degree.

In sum, because the focus of fiction is primarily on eliciting emotions, rather than on presenting factual information, fiction reading will be more likely influence empathy than non-fiction reading. The question remains, however, *how* fiction may influence empathy. Gerrig [12] argued that people may change as the consequence of fiction reading because they become fully immersed in a story, or in other words, they are transported into a narrative world. Gerrig [12] therefore presented the transportation metaphor to explain the effects of fiction on outcomes.

### The Role of Transportation in the Effects of Fictional Narratives

According to Gerrig [12], when people read a fictional narrative, they may become fully immersed into the story, which presents an alternative narrative world that is distant from the real world. While reading, people become transported into this narrative world, which often has been referred to a ‘being lost in a book’ [41]. Fiction can be an escape from the current world and by means of reading or watching, one is absorbed into the story told in the narrative. Transportation is defined as ‘a convergent process, where all mental systems and capacities become focused on events occurring in the narrative’ [14]. People lose track of time and fail to observe events going on around them; a loss of self-awareness may take place [42]. The narrative world is distant from the world in which the reader lives, and makes it possible that the events in the story are perceived as real within the story context, even when events would not be possible in reality [43].

The mental journey elicited by transportation makes it possible for readers to change as a consequence of reading fiction, because it elicits various processes, including emotional involvement in the story and identification with the characters [2], [3]. Many studies have shown that when readers become transported into a narrative, personal change is more likely to occur. For instance, Green and Brock [14] showed that when readers became transported into a story, their attitudes about topics that were included in the story changed more strongly than those who were not transported into a story. Similar findings were obtained in studies by Appel and colleagues who found that transportation into narratives are the main precursor of changes in the individual [33], [44], [45]. Although researchers have argued that transportation may refer to both cognitive and emotional involvement in a story, we propose that it is primarily through emotional transportation that people may change, because fictional narratives are primarily written to elicit emotions among the readers, such as fear, surprise or joy [2]. In sum, personal change is more likely to occur when a reader is emotionally transported into a story.

### Sleeper Effects of Fiction on Outcomes

Finally, in line with Appel and Richter [33], we expect that the effects of fiction experience on empathy are guided by an absolute sleeper effect [46], [47]. Absolute sleeper effects occur when the effects of a manipulation do not present themselves immediately, but manifest themselves over time. Absolute sleeper effects in fiction research assume that the effects of fiction reading on empathy will increase over time rather than present itself directly after the experience [33], [47]. There are two main reasons why these effects occur. First, Schank and Abelson [48] argue that when people organize information in stories (a process that fiction should facilitate, as it consists of stories already), the representations of these stories last better and longer. Thus, the effects of fiction should generally last longer than in logico-scientific mode of thinking (like in newspaper reports). Thus, when people are transported into fictional narratives, they are better in remembering the story, because they were more intensely involved in reading the story, which enables mental representations afterwards. Hence, fictional narratives as mental simulation of real world events [7] deepen the readers’ general tendencies to feel empathy with other people. Support for the idea that the effects of narrative fiction remains constant or may even increase over time comes from Paluck [49], who studied how a reconciliation radio program influenced perceptions of social norms in postwar Rwanda and found that through these radio stories, people’s perceived norms about how one should behave in social situations increased over time.

Second, for sleeper effects to occur, an incubation period is needed, in which people can rethink and relive that what has been read. Research on incubation has shown that spending some time on unrelated activities may enhance the effects of resolving problems, because an individual unconsciously connects the information from fictional narratives (e.g., people facing problems in their lives) with daily encounters, and consequently find new solutions through perspective taking and showing sympathy for other people [50], [51]. This process may occur both consciously and unconsciously. As an example of the unconscious influence of narrative fiction, Marsh et al. [10] showed that false statements from fictional stories were used by readers when they had to conduct a knowledge task one week after reading the story. Moreover, Appel and Richter [33] found that the influence of false statements in fictional stories on people’s beliefs increased over time. Therefore, we propose that the effects of fiction on empathy do not present themselves immediately but manifest themselves over time. To test this idea and show long term effects of fiction reading on empathy, in both experiments we measured empathy both directly after reading a fictional story and after a one-week delay.

### The Present Research

All in all, we expect that fiction will affect empathy over time only when a reader is emotionally transported into a story. The formal hypothesis of the study is:

Hypothesis 1: Fiction reading is positively related to empathy across time, but only when the reader is emotionally transported into the story.

To test the hypothesis, we present two studies in which the effects of fiction reading on empathy are investigated. In study 1, we investigated whether reading a Sherlock Holmes story influences empathy over the course of one week for readers who become emotionally transported into the story, while comparing these effects to non-fiction readers. In study 2, we sought to replicate these findings using a different story (a chapter from Blindness by Saramago), again using a control condition, while controlling for experienced negative and positive emotions. Stories were chosen because both stories include an event that happens to the main character (i.e., the murder to solve, and the spontaneous blindness of the man), and provided the opportunity to identify with the main characters, through which a reader was able to be transported into the story. Hence, readers could learn from the stories, as has been shown in research using these stories [52], [53]. Second, the authors are well-known and the stories would appeal a wide audience, and would not be appreciated only by a limited number of people who favor a particular genre. Moreover, stories were chosen for which Dutch translations were available, since we aimed to avoid any problems with translating the stories into Dutch (the language of the participants)

## Study 1

### Materials and Methods

#### Participants

Participants were 66 Dutch students who received course credits for participating in the study. They were randomly assigned to either the fiction or the control condition. All scales were measured using a self-report method. 36 participants completed the fiction condition, and 30 participants completed the control condition. There were no dropouts in the study; everyone who started the experiment finished it. Participants were on average 26 years old, 52% was female, and they spent on average 3.32 (SD = 3.88) hours per week on reading fictional books. We found no significant differences between the fiction and control condition in age, gender or amount of time weekly spent on reading fiction.

#### Procedure

Participants worked from home where they filled out the questionnaires and read the stories online via computer. The ECP (Ethische Commissie Psychologie/Ethical Commission Psychology) of the university where the study was conducted approved the consent procedure. All participants provided written informed consent of being participants in the study prior to participating in the study. The experiment leaders documented this digitally. All data were analyzed anonymously. Any information that could potentially lead to the identification of individuals (e.g., email-addresses, student registration numbers) was deleted after completing the study, and prior to the analyses. The study has been conducted according to the principles expressed in the Declaration of Helsinki. The same procedure was followed for Study 2.

All participants started by filling out some demographic variables, a range of study-irrelevant scales, and the empathy scale (T1). We included study-irrelevant scales (e.g., attitudes toward work measures) to hide the purpose of the study. Subsequently, participants were instructed to read either a few newspaper reports or a chapter from a fictional book. Participants read the fictional narrative (fiction condition) or a selection of articles from the Dutch newspaper *De Volkskrant* (control condition). After reading the text, they filled out the emotional transportation measure, the empathy scale, and some other irrelevant scales (T2). Participants had to provide a summary of what they had read, in order to check whether participants read the texts carefully. All participants provided accurate summaries, and hence no participant was deleted because of this reason. Precisely one week after reading the text, participants filled out a digital questionnaire from home, including the empathy scale (T3) and again irrelevant scales to avoid demand characteristics.

#### Text Material

In the fiction condition, participants read the first part of a short story from Arthur Conan Doyle, called ‘The Adventure of the Six Napoleons’ [54]. The story contained 2750 words and was read directly from the computer screen. The chapter was shown on one page, and readers could scroll-down to read the whole chapter. In the story, a plaster bust of Napoleon is shattered, a man is murdered and detective Sherlock Holmes is asked to solve the case. Participants did not read how Holmes solved the case.

In the control condition, participants read two stories from the Dutch high-quality newspaper *De Volkskrant*. The text was also around 2750 words long, and included a story about riots in Lybia and the nuclear disaster in Japan, which took place in March 2011. The stories were selected because they included experiences from individuals who were interviewed and followed during the riots in Lybia and disaster in Japan, and therefore would allow the reader to become emotionally transported into the non-fictional reports. The newspaper stories fitted the logico-scientific mode because the texts were primarily aimed at explaining events (what has happened), and why a particular event has happened. The newspaper reports were factual and focused on conveying information to the reader about a particular situation. Moreover, the nonfiction condition was not narrative in nature, but consisted of factual reports about real people. However, both conditions were matched in length and in content such that in both conditions, readers had the possibility to become transported into the text because individual people were central to the report or the story.

*Emotional Transportation* (α = .85) was measured directly after reading the text, using the scale from Busselle and Bilandzic [11]. It was measured with three items being: “The story affected me emotionally”, “During reading the text, when a main character succeeded, I felt happy, and when they suffered in some way, I felt sad”, and “I felt sorry for some of the characters in the text”. Answers could be provided on a 5-point scale (1 = not at all, 5 = to a very great extent). Transportation did not differ between the two conditions (F = 1.38, *df* 1,64, *ns*).

*Empathy* was assessed directly before the experiment (T1), directly after reading the text (T2) and one week after the experiment (T3), using the empathic concern scale of Davis [22], [23]. This subscale of Davis’ broader empathy scale was chosen because it reflected the part of empathy we were most interested in, being feeling sympathy and concern for others. It was measured using seven items, indicating the extent to which the participant feels empathic with other people. Example items are: “Sometimes I don’t feel sorry for other people when they are having problems” (reverse-scored), and “I am often quite touched by things that I see happen”. Davis found this scale to be valid and reliable [22], [23]. Reliability for the scale in this study was.71 at T1,.75 at T2, and.79 at T3. We also assessed whether transportation and empathy are distinct from each other. We ran a factor analysis and included the transportation items (measured at T2) and the empathy items (measured at T2). Two factors emerged, with the items loading on their corresponding factor, with item loadings all above.55 and no cross-loading of items on the other factors. The transportation scale correlated between.13 and.19 with empathy across the various time points. Hence, transportation and empathy represent two empirical different constructs.

We did not find gender differences in transportation (F = .36, *df* 1,64, *ns*). In our analyses, we controlled for the influence of difficulty of the texts. At T2 (directly after reading the stories) we measured whether participants understood the stories they read through the *Narrative Understanding* scale [11] (α = .76). Example items of this scale are: “At points, I had a hard time making sense of what was going on in the stories” and “My understanding of the character is unclear” (both items recoded). Moreover, we also measured at T2 the extent to which readers were able to focus their attention to the stories, through the *Attentional Focus* scale [11] (α = .91). Examples are: “I found my mind wandering while I was reading the story” and “I had a hard time keeping my mind on the stories” (both items recoded). We found that the fictional story was significantly higher in attentional focus (F = 5.05, *df* 1,64, *p*<.05) while narrative understanding was not significantly different between the two conditions, indicating that the fictional story was easier to focus the attention to. We added these scales to the regression analyses in order to control for the alternative explanations that that readers who have more difficulty in understanding the text or to focus their attention to the text will be less likely to change in empathy over time.

### Results and Discussion

Hierarchical regression analyses were used to test the hypotheses. To reduce multicollinearity bias, emotional transportation was first standardized before interaction terms were calculated [55]. In the first step, empathy T1 and the control variables were added to the equation, after which in two separate steps, the independent variables and the interaction term were added. For significant interactions, slopes were calculated for the two experimental conditions. Table 1 presents the variable means and the correlations among the variables under study. Standardized coefficients (betas) are reported for the regression analyses in order to be able to compare effect sizes with other predictors in the model, while unstandardized coefficients (B’s) are reported for interactions in order to ascertain strength of the effect [55]. To show the sleeper effect, we estimated the effects of transportation on empathy both immediately (T2) and after one week (T3).

**Table 1:** Study 1: Means, standard deviations, reliabilities and correlations of the study variables.

	Variable	*Time*	*M*	*SD*	1	2	3	4	5	6	7
1	Condition	T1	.55	–	–						
2	Empathy	T1	3.61	.61	−.15	**.71**					
3	Narrative Understanding	T2	3.74	.91	.18	−.01	**.76**				
4	Attentional Focus	T2	3.41	1.14	.27*	.04	.70**	**.91**			
5	Emotional Transportation	T2	2.21	.91	−.15	.13	−.01	.14	**.85**		
6	Empathy	T2	3.54	.64	−.27*	.91**	−.08	−.03	.16	**.75**	
7	Empathy	T3	3.56	.59	−.14	.85**	−.07	.07	.19	.87**	**.79**

*Note. Reliabilities are reported along the diagonal. N* = 66; Condition: 0 = Control, 1 = Fiction. *p<.05, **p<.01.

**Figure 1:**
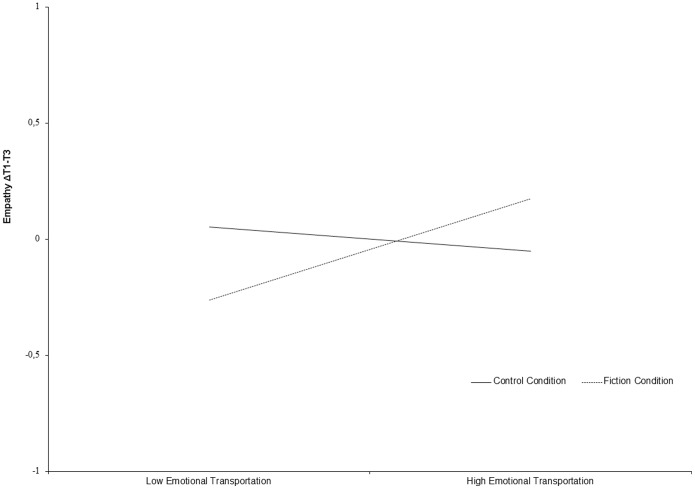
The interaction pattern between emotional transportation and condition in relation to changes in empathy from T1 to T3 (Study 1).

Table 2 presents the results of the hierarchical regression analyses. As predicted, there was no immediate effect of the interaction between condition and transportation on empathy T2 (*β* = .03, *ns*). Empathy T1 was a strong predictor of empathy T3 (*β* = .81, *p*<.001). Narrative understanding (*β* = −.16, *ns*), attentional focus (*β* = .14, *ns*), condition (*β* = −.04, *ns*), and emotional transportation (*β* = −.09, *ns*) were unrelated to empathy T3. However, in line with the study hypothesis, the interaction between emotional transportation and condition was significantly related to empathy T3 (*β* = .17, *p*<.05). Figure 1 presents the interaction pattern in relation to the change in empathy from T1 to T3. The relation of emotional transportation with empathy T3 was positive and significant for fiction readers (unstandardized slope B = .09, *p*<.05), and not significant in the control condition (B = −.02, *ns*). Further analyses revealed that especially under conditions of low transportation, empathy differed significantly between the two conditions (*p*<.10), but with increasing transportation, empathy increased for fiction readers while it was not significant for the control condition. We also calculated regions of significance for the effects of emotional transportation on changes in empathy [56]. Regions of significance indicate between which values of emotional transportation the impact on change in empathy is significantly different between the two conditions. It was found that the region of significance ranged between −21.05 and −1.35 of the standardized score of transportation. These values are outside the range of the standardized transportation score (−1.33 to 1.97). Hence, while the relationship was non-significant for nonfiction readers, the relationship was positive for fiction readers along every point of the slope. This indicates that for low transportation, empathy of fiction readers became significantly lower than of nonfiction readers, and for highly transported readers, empathy significantly increased over time. Therefore, our hypothesis is supported; fiction readers become more empathic over the course of a week when they are emotionally transported into the story, while lowly transported fiction readers became less empathic over time. As expected, this was not the case in the control condition.

**Table 2:** Study 1: Hierarchical regression analyses predicting empathy T3.

	*Empathy T2*			*Empathy T3*		
	Step 1	Step 2	Step 3	Step 1	Step 2	Step 3
*Control variables*						
Empathy T1	.91***	.89***	.88***	.84***	.83***	.81***
Narrative Understanding	−.05	−.05	−.04	−.17	−.16	−.16
Attentional Focus	−.03	−.01	−.01	.16	.17	.14
*Independent variables*						
Condition		−.12	−.12		−.06	−.04
Emotional Transportation		.04	.02		.02	−.09
*Interaction*						
Emotional Transportation * Condition			.03			.17*
F	100.19***	65.03***	53.44***	54.08***	32.00***	28.09***
ΔF	100.19***	2.93	.14	54.08***	.43	3.95*
R^2^	.83	.84	.85	.73	.73	.75
ΔR^2^	.83	.02	.00	.73	.00	.01

*Note*. *N = *66. Standardized regression coefficients are reported. Condition: 0 = control; 1 = fiction. *p<.05, **p<.01, ***p<.001.

Study 1 provides first evidence that fiction reading causes empathic skills to increase over time when the reader becomes emotionally transported into the story, while the reverse occurs when the fiction reader does not become transported at all: then the reader actually becomes less empathic. Hence, when people read a Sherlock Holmes story and become fully engaged in the story and identify strongly with the main characters, empathy is enhanced over time and empathy decreases for non-transported readers. To further test the hypothesis that fiction reading can influence empathy and to cross-validate the findings, we conducted a second study. In this study, we used another fictional story to ascertain whether the effects hold across fictional stories and genres. Moreover, the question is whether the effects of transportation into fiction experience cannot be attributed to the emotions people experience while reading a fictional text. Therefore, in analyzing the effects of emotional transportation on change in empathy over one week, we now controlled for experienced negative and positive emotions, in order to rule out the possibility that it is only the emotions people experience after reading that changes their empathic skills [7].

## Study 2

### Materials and Methods

#### Participants

Participants were 97 undergraduate Dutch students who received course credits for participating in the study. They were randomly assigned to either the fiction or the control condition. All scales were measured using a self-report method. Fifty participants completed the fiction condition, and 47 participants completed the non-fiction condition. There were no dropouts in the study. None of the participants from study 1 could participate in this study. Before reading the text, age, gender (1 = male; 2 = female) and narrative experience were measured. On average, participants were 24 years old, and 74% were female. Narrative experience was rated by the average amount of fictional books one reads annually. On average, participants read 13 fictional books per year.

#### Procedure

Participants worked again from home where they filled out the questionnaires and read the stories online via the computer. All participants started by filling out some demographic variables, a range of study-irrelevant scales, and the empathy scale (T1). Subsequently, participants read the fictional narrative (fiction condition) or a selection of articles from the Dutch newspaper *NRC Handelsblad* (control condition). After reading the text, they filled out the emotional transportation measure as well as the narrative understanding and attentional focus measures, the empathy scale, and some other irrelevant scales, such as engagement in leisure activities, attitudes about work and creativity, in order to avoid demand characteristics (T2). Furthermore, participants were asked to give a summary of what they had read. The first author and two colleagues assessed whether participants gave accurate summaries. Since all of the participants provided accurate summaries, none of the responses were deleted because of inaccurate reading of the text. Precisely one week after reading the text, participants filled out a digital questionnaire from home, including the empathy scale (T3).

#### Text Material

In the fiction condition, participants read the first chapter from Nobel Prize winner José Saramago’s *Blindness* [57], [58]. A Dutch translation of the chapter was used for the study, since all of the participants were native Dutch citizens. Work from a Nobel Prize for Literature (1998) winner was selected because this work would appeal to many readers. The chapter describes a man who, while in his car waiting for the traffic lights, spontaneously becomes blind. Passengers bring the man to his home, while another man, who promises to bring his car home, steals it. When the man is home, he falls asleep and dreams. When his wife returns home, she brings him to an ophthalmologist, who is not able to diagnose his condition (end of chapter). While being fictional, the chapter contains a strong emotional component, through picturing the man who instantly becomes dependent upon other people when turning blind. The chapter contains 5330 words, and was read directly from the computer screen. The chapter was shown on one page, and readers could scroll-down to read the whole chapter. In the control condition, participants read parts of the Dutch newspaper *NRC Handelsblad*. It was 5220 words long, and included in total five stories (e.g., about riots in Greece and liberation day in the Netherlands). Again, newspaper articles were selected that included stories about individual people and therefore provided means to become transported into the stories. Participants read the articles directly from the computer screen.

*Emotional Transportation* was measured directly after reading the text, using the same scale as study 1 [11]. Reliability of this scale was.87. Transportation was higher in the fiction condition than the nonfiction condition (F = 13.56, *df* 1,95, *p*<.001). *Empathy* was assessed directly before the experiment (T1), directly after reading the text (T2) and one week after the experiment (T3), using the same scale as in study 1 [22]. Reliability for the scale in this study was.75 at T1,.79 at T2, and.77 at T3.

We controlled for age, gender and narrative experience, narrative understanding (α = .67), and attentional focus (α = .92). We did not find gender differences in transportation (F = .13, *df* 1,95, *ns*). Narrative understanding and attentional focus were measured at T2 using the same scales as in study 1 [11]. We found that the fictional story was easier to understand and to focus the attention to (narrative understanding: (F = 9.80, *df* 1,95, *p*<.001; attentional focus: F = 7.03, *df* 1,95, *p*<.01). We also controlled for positive and negative emotions. These two were measured directly after reading the text (T2), with scales from Djikic et al. [28]. Participants were asked to rate for eight emotions the extent to which they felt these emotions after reading the text. Positive affect was measured with four items (happiness, contentment, excitement, and awe; α = .80). Negative affect was also measured with four items (sadness, anxiety, anger, and fearfulness) and was found to be reliable (α = .74).

#### Analysis

Hierarchical regression analyses were used to test the hypothesis. To reduce multicollinearity bias, independent variables were first standardized before interaction terms were calculated [55]. In the first step, control variables were added to the equation, after which in two separate steps, the independent variables and the interaction term were added. For significant interactions, slopes were calculated for the two experimental conditions. Table 3 shows the means of the variables and the correlations of the variables under study. As expected, age was positively correlated with narrative experience (*r = *.24, *p*<.05), and gender was also positively related to narrative experience (*r = *.22, *p*<.05), indicating that women on average read more fictional books than men. Gender was also positively related to empathy T1 (*r = *.35, *p*<.01), empathy T2 (*r = *.31, *p*<.01), and empathy T3 (*r = *.38, *p*<.01), indicating that women on average rated their empathic skills to be higher than men, which is consistent with previous studies by for instance Mar et al. [7].

**Table 3:** Study 2: Means, standard deviations, reliabilities and correlations of the study variables.

	Variable	*Time*	*M*	*SD*	1	2	3	4	5	6	7	8	9	10	11	12
1	Age	T1	23.75	9.62	–											
2	Gender	T1	1.74	–	−.16	–										
3	Narrative Experience	T1	13.48	17.61	.24*	.22*	–									
4	Condition	T1	.52	–	.03	−.10	−.05	**–**								
5	Empathy	T1	3.61	.53	.00	.35**	.22*	−.10	**.75**							
6	Narrative Understanding	T2	3.19	.83	.03	−.19	−.09	.31**	−.06	**.67**						
7	Attentional Focus	T2	3.09	1.04	.01	−.11	−.12	.26**	−.09	.87**	**.92**					
8	Emotional Transportation	T2	2.92	.89	.04	−.04	−.08	.35**	.16	.42**	.42**	**.87**				
9	Positive Affect	T2	3.54	1.22	−.11	.10	−.13	−.38**	.11	−.05	−.02	.20	**.80**			
10	Negative Affect	T2	3.37	1.28	−.04	.08	−.02	.19	.09	.11	.12	.38**	.33**	**.74**		
11	Empathy	T2	3.58	.52	−.06	.31**	.21*	−.14	.90**	−.05	−.05	.20*	.18	.16	**.79**	
12	Empathy	T3	3.54	.52	−.07	.38**	.18	−.18	.85**	−.15	−.14	.12	.23*	.21*	.86**	**.77**

Note. Reliabilities are reported along the diagonal. N = 97; *p<.05, **p<.01. Condition: 0 = Control, 1 = Fiction.

### Results and Discussion

**Figure 2:**
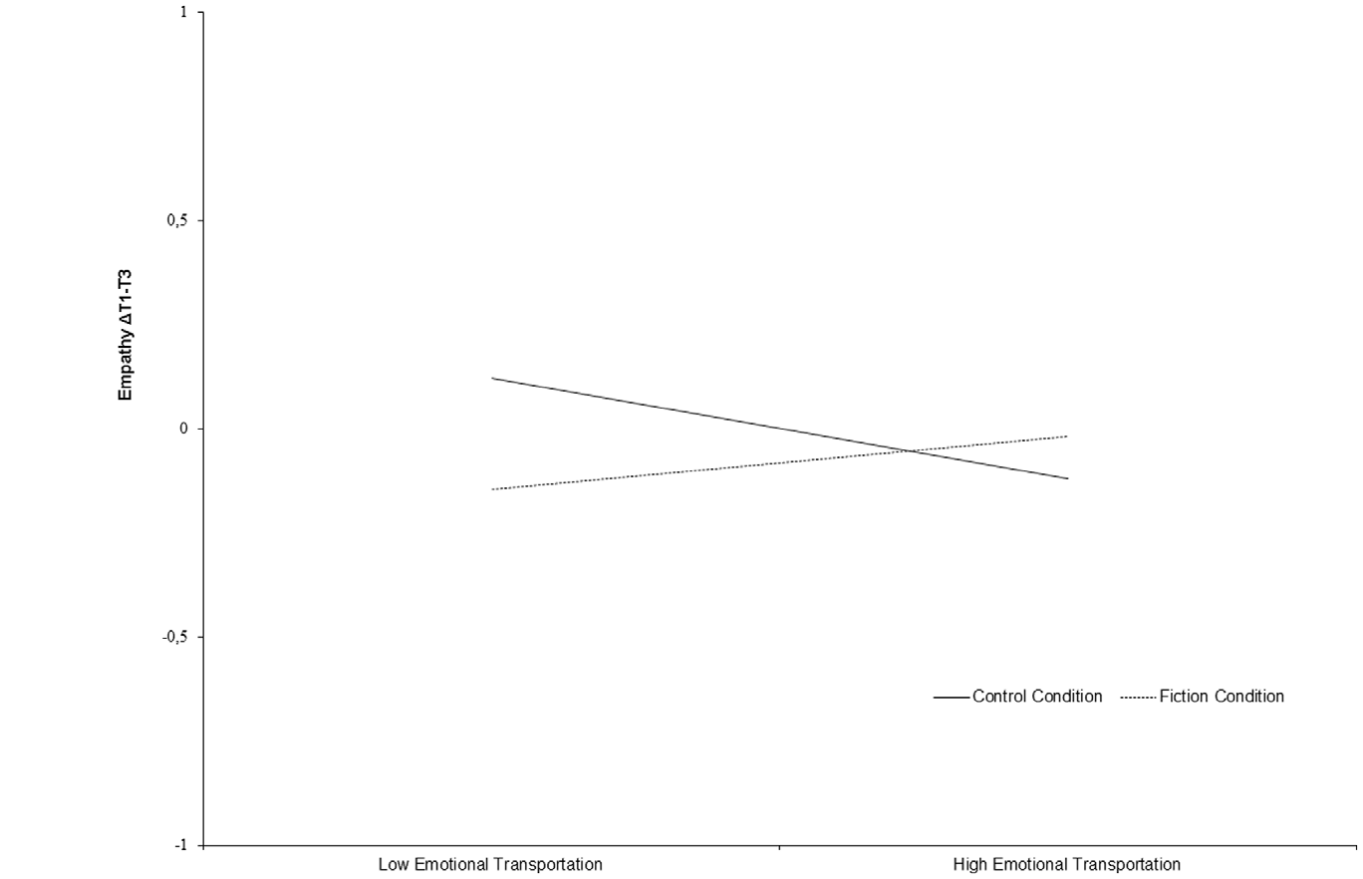
The interaction pattern between emotional transportation and condition in relation to changes in empathy from T1 to T3 (Study 2).

Hierarchical regression analyses are shown in Table 4. The interaction between condition and transportation was not related to empathy T2 (*β* = .04, *ns*). Neither narrative understanding (*β* = .04, *ns*), attentional focus (*β* = −.12, *ns*), condition (*β* = −.05, *ns*), nor emotional transportation (*β* = −.12, *ns*) were significantly related to empathy T3, while controlling for age, gender, narrative experience, stability of empathy (*β* = .82, *p*<.001), and positive and negative affect. The interaction term between condition and emotional transportation was significant (*β* = .18, *p*<.05; ΔR^2 = ^.01). The interaction term in relation to changes in empathy from T1 to T3 is graphically represented in Figure 2. The slope for non-fiction readers was negative (B = −.12, *p*<.05), while the slope for fiction readers was positive (B = .07, *p*<.05). Similar to study 1, at low levels of transportation the two conditions were significantly different (*p*<.05), but the effects of transportation were opposite in the two conditions. We estimated a region of significance *outside* −.38 to 25.03. Given the range of the standardized score of transportation from −2.16 to 2.35, it can be concluded that at low levels of transportation, fiction readers became lower in empathy over time, and when transportation increased somewhat, empathy increased as well, while for nonfiction readers who were low in transportation, the effect was negative when they became more transported. Thus, the study hypothesis is partially supported in study 2; emotional transportation in fiction reading influences empathy over time, but only when fiction readers have low levels of transportation become less empathic.

**Table 4:** Study 2: Hierarchical regression analyses predicting empathy T3.

	*Empathy T2*			*Empathy T3*		
	Step 1	Step 2	Step 3	Step 1	Step 2	Step 3
*Control variables*						
Age	−.07	−.08	−.07	−.04	−.04	−.02
Gender	−.05	−.04	−.04	.05	.05	.07
Narrative Experience	.05	.05	.05	.01	.00	−.01
Empathy T1	.89***	.88***	.88***	.81***	.81***	.82***
Positive Affect	.06	.02	.02	.09	.06	.09
Negative Affect	.07	.08	.07	.12	.14*	.12
Narrative Understanding	.12	.11	.11	.05	.05	.04
Attentional Focus	−.10	−.10	−.10	−.14	−.12	−.12
*Independent variables*						
Condition		−.08	−.07		−.06	−.03
Emotional Transportation		.06	.03		−.03	−.17
*Interaction Effect*						
Emotional Transportation * Condition			.04			.18*
F	51.56***	41.56***	37.50***	36.91***	29.41***	28.26***
ΔF	51.56***	1.10	.30	36.91***	.64	4.56*
R^2^	.82	.83	.83	.77	.77	.79
ΔR^2^	.82	.00	.00	.77	.00	.01

*Note*. *N = *97. Standardized regression coefficients are reported. Gender: 1 = male, 2 = female; Condition: 0 = control; 1 = fiction. *p<.05, **p<.01, ***p<.001.

In sum, while we found that in study 1, empathy was enhanced over a period of one week, in study 2, we found that low transportation led to lower empathy over time. Inspection of the interaction effects revealed that especially at low levels of transportation, empathy became lower among fiction readers. However, since transportation is a continuous variable, increase of transportation only enhances empathy for fiction readers, and not for non-fiction readers. Moreover, these effects could not attributed to difficulty of the texts, or experienced negative or positive emotions. Although the regression analyses showed that when people experienced negative emotions while reading, the interactions of condition and transportation were also significant, showing that fiction reading influences empathic skills beyond simple emotional effects and this can be both negatively and postively.

## General Discussion

The current study investigated the influence of fictional narrative experience on empathy over time. In two experimental studies, we were able to show that self-reported empathic skills significantly changed over the course of one week for readers of a fictional story by fiction authors Arthur Conan Doyle or José Saramago. More specifically, highly transported readers of Doyle became more empathic, while non-transported readers of both Doyle and Saramago became less empathic. These effects were not found for readers in the control condition in both studies, although nonfiction readers in study 2 decreased in empathy when transportation increased. Increase of emotional transportation enhances empathy for fiction readers while it does not for nonfiction readers, such that it leads to higher empathy at relatively high levels of transportation. For study 1, indeed high transportation led to increases in empathy for fiction readers, while for both studies 1 and 2 absence of transportation was associated with decreases in empathy for fiction readers. This could be explained because when a reader is not able to identify with a text and does not become transported, this might lead to disengagement, with the reader being distracted and frustrated, as suggested by Pelowski and Akiba [29]. In other words, a reader has to become fully transported into the story to change as a consequence of reading, to become more empathic. When a reader is not able to identify with a fictional narrative and does not become transported, this might lead to disengagement, with the reader being distracted and frustrated. When readers disengage from what they read, they possibly become more self-centered and selfish in order to protect the sense of self in relation to others [17]. Yet, these results are important, because previous research has claimed that fiction reading has positive effects [6]–[7], while we are amongst the first who also show that fiction reading might have negative effects, when readers do not become transported, and hence, disengage from literature.

For the participants in study 2, empathic skills decreased somewhat when they became emotionally transported into the newspaper stories. Finally, from study 2 we conclude that these effects hold even after controlling for factors such as general narrative experience, experienced negative and positive emotions during reading and the experienced difficulty of the texts. Therefore, the effects of increased empathic skills cannot be solely attributed towards the emotions people experience in response to either a fictional or non-fictional text or the difficulty people have in reading a texts.

These are the first empirical studies showing under realistic conditions that fiction reading is related to empathic skills. Although previous studies have pointed towards these effects [6], [7], we show that reading real stories relates to how people sympathize with others, are able to take multiple perspectives, and feel for unfortunate others. Increase of empathy is important for people because empathy is positively related to creativity [26], performance at work [25], and prosocial and cooperative behaviors [59], [60].

### Research Implications

The current study has a number of implications for future research on the role of fictional narrative experiences. First, and most importantly, the current study followed the transportation framework of Gerrig [12], [17] to postulate specific predictions of the conditions under which fiction experience relates to outcomes. We have shown that emotional transportation influences the reactions toward fiction reading in terms of changes in empathy. Since the main effects of the conditions were not significantly related to change of empathic skills over time, it is not the activity of reading itself that transforms the self, but the emotional involvement in a narrative [28], [45]. Thus, this study adds to the recent empirical findings that it is transportation that influences whether people’s beliefs about the world are influenced [28]. Therefore, it is imperative for future research on the effects of fictional narrative experience to take the role of transportation processes into account. We have argued that it is through sympathizing with the characters in a story that people become more empathic. However, not every fictional narrative will provoke sympathy; for instance characters in a story may act in ways that the reader disapproves, and consequently no sympathy is felt for the characters. It might be possible that other effects of these experiences of disapproval are established, such as changes in moral values [44]. This study also corroborates this hypothesis by showing that low transportation leads to lower empathy over time. Future research may shed more light on this issue.

Moreover, the study has shown that effects of fictional experience are different from the control condition in which non-fictional texts were used [12]. Although both types of narratives may elicit strong emotions, and people may become engaged in reading both types of narratives [15], the outcomes may be opposite to each other. While transportation into fiction may cause people to sympathize with other people, through felt emotions, high involvement and sympathy for people in non-fiction stories may create felt obligations to do something while not possible, which consequently leads to lower empathy [39], [40]. When we read non-fiction, readers have to suspend disbelief to be changed by the story. When reading fiction, however, disbelief has not to be suspended because readers are likely to accept information from fiction without asking themselves whether the information is true or not [12]. Therefore, the processes through which fiction experience relate to outcomes is wholly different from more logical processes, which are guided by non-fiction reading [16]. Future research should further disentangle the differential impact of these fictional and non-fictional narrative experiences.

Finally, the current study has shown that the effects do not present themselves immediately, but that the effects are guided by an *absolute sleeper effect* [33]. Theoretically, fictional narratives are more likely to influence behavior over the course of a week rather than directly after the narrative experience, because the process of transformation of an individual needs time to unfold [38], [44]. For instance, people think back and mentally relive the story they have read. The effects of fictional narrative experience may flourish under conditions of an incubation period, in which the changes in empathy become internalized and part of the self-concept [29]. Therefore, research on fictional narrative experience should be guided by a temporal design of the proposed effects. For instance, if the proposed outcomes of fictional narrative experiences are experienced emotions or psychological detachment from work, the effects will be more immediate and direct rather than when outcomes such as empathy or creativity are investigated.

### Limitations and Suggestions for Further Research

One of the limitations of the current study was that the participants in the fiction condition only read the first part of a Sherlock Holmes story and the first chapter of the novel by Saramago. Therefore, it is possible that the effects of the fictional narrative experience are somewhat underestimated, since the experience of a complete story or novel may be different than reading a single chapter. First, if empathy is positively related to experience with reading fictional narratives, as previously suggested [6], [7], then it can be expected that longer exposure to a novel will have stronger effects than reading a single chapter. Furthermore, readers of a fictional narrative can identify with the main characters [12], [14], and such identification and sympathizing with the main characters can be expected to be stronger as the reader becomes more familiar with them, in other words, when one reads more of a novel. Thus, the effects of fictional narrative experiences may be stronger as one has more prolonged exposures. However, it might be also the case that because participants in the control condition read multiple stories, even though they had more opportunities to become transported, these opportunities were less expanded than in the fiction condition. Future research should therefore include more similar stories to ascertain the effects of fiction and non-fiction. For instance, nonfictional reports could be constructed which are equivalent to fictional stories, such that more specific evidence can be gathered concerning the impact of fiction reading on outcomes.

A limitation to the beneficial effects of fictional narrative experiences on perceived empathy could be that there are ceiling effects regarding increases of empathy following a fictional narrative experience. That is, although we have shown that empathy increases over the course of one week when one becomes transported into a narrative, it might be the case that the potential effects become smaller for avid readers or for highly empathic people. The sample of the current study consisted mainly of younger randomly selected students, who may therefore be more likely to be influenced by fictional narratives, than groups of highly experienced readers or a selection of highly empathic people. However, whether this line of thought is actually true remains an empirical question. In contrast, low transportation may lead to disengagement from a text. When readers have to read a text, they may feel less empathy with other people when they cannot identify with the characters in the text, and they may experience feelings of rejection, disgust, and disengagement. Hence, their empathic skills may decrease when they disengage.

A related question pertains to what happens during the week that is between reading a text and increase in empathy. Future research should investigate how the process evolves over time, so that better knowledge is gained as to what exactly happens over time when people have read and are transported into fictional stories.

An interesting avenue for further research is to investigate other outcomes of fictional narrative experiences. Next to affecting empathic skills of the reader, fictional narrative experiences may also influence creativity [17], psychological detachment and recovery from work. Because fictional narrative experience is closely linked towards imaginative processing, readers of fiction learn to develop imagination in alternative worlds, through transportation in narratives. Subsequently, people develop broader action repertoires, causing them to be more creative in finding solutions for complex problems [17]. Moreover, through fiction experience, people take the opportunity to relax and unwind from work through which they can recover from their work. In contrast, non-fiction reading might be associated with alternative consequences than empathy. For instance, reading about events that have taken place in reality may create feelings of guilt and obligation [39]. Future research should investigate these alternative outcomes as well.

Another area for future research is to investigate the differential roles of transportation processes in determining outcomes. In the current study, we have proposed that emotional transportation will influence the extent to which people’s empathy is changed over time. Because fiction experiences are inherently emotional in nature [2], it is the emotional engagement in the story and the characters in the story that cause people to identify and sympathize with others. However, if people just want to know how a story ends and how a mystery is solved, and hence are only cognitively transported without being emotionally involved [12] other outcomes may be expected, such as enhanced problem solving skills. Hence, depending on the outcome of fictional narrative experiences, the type of transportation (i.e., emotional or cognitive) may matter highly in predicting the outcomes.

A related area is the increasingly blurred distinction between fiction and non-fiction. In the current study, we used for the control condition articles from a newspaper, belonging in the nonfiction category or logico-scientific thinking [16]. Recently, however, an increasing number of books are published that are based on actual events, but yet are written in ways very similar to fictional novels, such that they may be very narrative in nature, in which the author in detail describes how events affected people’s thinking and emotions (a genre claimed to have started with Truman Capote’s *In Cold Blood*). Hence, the fictional nature of these types of stories (i.e., the author stresses believability of the story, and the narrative primarily aims at eliciting emotions in the reader), may constitute a fictional narrative experience for an individual, and hence effects may occur in line with the transportation framework [17]. Therefore, the fictional boundaries of non-fictional stories become broader, offering the potential experiences of fictional narratives, including the effects attributed to such experiences.

Finally, in the current study we have used self-reports to measure participants’ empathic skills. Therefore, we relied on how people assess how empathic they are. Although for future research it is recommended to obtain multiple perspectives on the outcomes under study (e.g., peer-ratings of empathy or actual empathic behavior), for the current study it was deemed appropriate to use self-reports, because we were mainly interested in individual *change* in empathy as well as the moderating role of transportation. As previous research has shown, common method bias is less likely to affect moderated hypotheses [61].

### Conclusion

The current study investigated how fictional narrative experience relates to empathic skills over time. Through two experiments, it was shown that transportation into fictional narratives influence empathy over time; a lack of transportation is related to lower empathy, while a high level of transportation might be related to higher empathy. The study shows that fictional narrative experiences have effects on people’s skills, such as empathy.
